# Scientific dishonesty—a nationwide survey of doctoral students in Norway

**DOI:** 10.1186/1472-6939-14-3

**Published:** 2013-01-05

**Authors:** Bjørn Hofmann, Anne Ingeborg Myhr, Søren Holm

**Affiliations:** 1 Centre of Medical Ethics, University of Oslo, Oslo, Norway; 2 Department for Health, Technology and Social Sciences, University College of Gjøvik, Gjøvik, Norway; 3 Genøk-Centre for Biosafety, The Science Park, Tromsø and Faculty of Health Sciences, University of Tromsø, Tromso, Norway; 4 Centre for Social Ethics and Policy, School of Law, University of Manchester, Manchester, UK

**Keywords:** Dishonesty, Fabrication, Falsification, Plagiarism, Misconduct

## Abstract

**Background:**

The knowledge of scientific dishonesty is scarce and heterogeneous. Therefore this study investigates the experiences with and the attitudes towards various forms of scientific dishonesty among PhD-students at the medical faculties of all Norwegian universities.

**Method:**

Anonymous questionnaire distributed to all post graduate students attending introductory PhD-courses at all medical faculties in Norway in 2010/2011. Descriptive statistics.

**Results:**

189 of 262 questionnaires were returned (72.1%). 65% of the respondents had not, during the last year, heard or read about researchers who committed scientific dishonesty. One respondent had experienced pressure to fabricate and to falsify data, and one had experienced pressure to plagiarize data. On average 60% of the respondents were uncertain whether their department had a written policy concerning scientific conduct. About 11% of the respondents had experienced unethical pressure concerning the order of authors during the last 12 months. 10% did not find it inappropriate to report experimental data without having conducted the experiment and 38% did not find it inappropriate to try a variety of different methods of analysis to find a statistically significant result. 13% agreed that it is acceptable to selectively omit contradictory results to expedite publication and 10% found it acceptable to falsify or fabricate data to expedite publication, if they were confident of their findings. 79% agreed that they would be willing to report misconduct to a responsible official.

**Conclusion:**

Although there is less scientific dishonesty reported in Norway than in other countries, dishonesty is not unknown to doctoral students. Some forms of scientific misconduct are considered to be acceptable by a significant minority. There was little awareness of relevant policies for scientific conduct, but a high level of willingness to report misconduct.

## Background

*Scientific dishonesty* frequently refers to actions or omissions in connection with research, which leads to false or distorted scientific results or gives misleading information about an individual contribution to research [1-3]. Scientific dishonesty is problematic for a number of reasons. It may directly or indirectly harm vulnerable research participants. It may undermine the general trust in science and scientists, and it may cause harm if future research or therapy attempts to rely on fraudulent results.

Traditionally the norms of science are learned by witnessing exemplary behaviour [4]. Mentors, supervisors and institutions played a significant role in promoting such norms. This has changed significantly as the number of researchers and time pressure has increased, as research has become international and interdisciplinary, as there are tight ties between academia, private industry, and governmental research agencies, and as there is an experienced increase in pressure for publications and achieving grants. Although only the serious cases grab the media headlines, such as the Sudbø case in Norway [5], it remains important to be aware of other types of questionable behavior that threaten the integrity of science [6,7].

How widespread is scientific dishonesty? This is a difficult question with significant methodological challenges [8], and divergent answers can be found within empirical research [9-11]. Not surprisingly there seems to be significant underreporting of dishonesty and misconduct [12,13]. A systematic review and meta-analysis [11] showed that an average of 2% of scientists admitted serious forms of misconduct (fabrication, falsification or modification of data or results) at least once. Up to 34% admitted other questionable research practices, and in surveys asking about the behaviour of colleagues, ‘admission’ rates were 14% for falsification, and up to 72% for other questionable research practices. Medical/pharmacological researchers reported misconduct more frequently than others, although not significantly so, and it is concluded by the author that it is likely that the result is a conservative estimate of the true prevalence of scientific misconduct [11]. In a Nordic context population based calculations indicate that Denmark has 1–2 cases/million inhabitants per year, Finland 1–2 cases/million per year, and Norway 1 case/million per year [14].

It is argued that there is no clear relationship between attending courses in research ethics or mentoring and scientific dishonesty. Training has not been shown to be effective in preventing problematic behaviour, and mentoring both increased and decreased the likelihood of problematic behaviours, depending on the kind of mentoring (research, financial, survival, personal, ethics) [15].

A study of undergraduate and postgraduate pharmacy students revealed widespread deficiencies in student knowledge of, and attitudes towards, plagiarism [16]. Many students did not perceive plagiarism as a serious issue and the use of inappropriate strategies for sourcing and acknowledging material was common. Similar results are found elsewhere [17].

Although medical students consider dishonest behaviour to be wrong, a substantial number of students report that they engage in dishonest behaviour [17,18]. Students’ attitudes appear to be stable throughout the years of the study [19], and their willingness to cheat may be culturally dependent and starts before medical school [20]. The baseline knowledge of responsible conduct of research for biomedical sciences students is shown to be inadequate and inconsistent [21].

There appear to be several motives for scientific dishonesty: the universities’ admission requirements, the system for financing, access to scholarships [3], and the perceived need for personal progress and success.

Scientific dishonesty has been studied in the Nordic countries [22-25], but our knowledge is still limited and of medium or poor quality. One study has been carried out in Sweden, [3] but comparative knowledge is necessary to assess the extension of dishonesty and postgraduate students’ attitudes towards scientific dishonesty.

What are PhD-students experiences with scientific dishonesty, what are their attitudes towards various forms of scientific dishonesty, and what do they know about regulations and policies? These are the key questions of our survey carried out among PhD-students at medical faculties in Norway.

## Methods

A two-page questionnaire combining a survey developed at the Department of Medical Ethics in Lund, Sweden [3] with a survey developed by Kalichman was applied [9,26]. Terms such as ‘scientific dishonesty’, ‘plagiarism’, ‘fabrication of data’, and ‘falsification’ were given standard definitions in the introduction. The questionnaire was piloted with a group of students similar to the intended respondents, and a few ambiguous statements were identified and re-written.

The participants in the study were post-graduate students being enrolled in PhD-programs at all medical faculties in Norway, i.e., at the universities in Oslo, Bergen, Trondheim, and Tromsø. The questionnaires were distributed to doctoral students attending basic, obligatory courses in research methodology, philosophy of science, and research ethics at the four medical faculties in Norway in the fall 2010 and in the winter 2011 in Tromsø and Trondheim. There were two courses in Oslo in the fall 2010, one of the courses was held in Norwegian (Oslo 1) and one in English (Oslo 2). The questionnaires were anonymous and participation was voluntary. The questionnaires were distributed and collected before any discussion of scientific misconduct had taken place in the course. Answer categories were Yes/No/Uncertain, and Strongly Disagree/Disagree/Neither Agree nor Disagree/Agree/Strongly Agree.

Descriptive statistics was used to find differences in post graduate students’ attitudes towards and experiences with scientific dishonesty. Categorical variables were compared using the Student *t* test, Fisher exact test, Mann–Whitney test (p < 0.05 was considered significant).

Answers were not traceable to participants. The project is registered at the Norwegian Social Science Data Service, overseeing privacy and data protection according to the Personal Data Act.

## Results

The total number of questionnaires distributed and returned at the various universities, together with the participants’ answers to questions about academic background is displayed in Table 1. 262 questionnaires were distributed, of which 189 were returned, giving an overall response rate of 72.1%. The doctoral candidates’ voluntary participation and anonymity were emphasised in the covering letter and when handing out the questionnaires.

**Table 1:** Total number of questionnaires distributed and returned, together with the participants’ answers to questions about academic background

**Site: Questions**	**Bergen**	**Oslo 1**	**Oslo 2**	**Tromsø**	**Trondheim**	**All in Norway**	**All in Sweden**
Returned/distributed (n)	38/56	47/48	31/39^†^	32/39	41/80	189/262	134/230
Response rate (%)	67,9	97,9	79,5	82,1	51,3	72,1	58,3
Undergraduate studies in Norway n (%)	27 (71)	39 (83)	15 (47)	25 (78)	31 (76)	137 (72)	-
Doing Clinical/Basic/Other research	20/11/6	24/12/10	7/18/6	14/8/10	20/5/16	85/54/48	-
Years of experience: <1yr/1-2yrs/>2yrs	23/11/4	34/9/4	17/8/6	11/15/6	33/7/1	118/50/21	-
Lectures or courses in science ethics as part of undergraduate studies (Yes/No/I do not remember)	21/12/5	31/11/5	25/4/2	22/7/3	25/12/4	124/66/20	-
Obligatory course (Yes/No)	YES	YES	YES	YES	YES	262/262^‡^	128/6
Obligatory exam (Yes/No)	YES	YES	YES	YES	YES	262/262	91/43

Data from Sweden reproduced from Nilstun 2010.

^†^One was returned blank (and is not counted in the response rate as it does not contribute with information).

^‡^The doctoral courses covering science ethics were obligatory at all universities in Norway, but the participation in the teaching every day was not obligatory.

The response rate varied greatly between the five Norwegian universities—from about 51% (Trondheim) to 98% (Oslo). Most of the respondents had taken their undergraduate studies in Norway (72%), except for the course in Oslo given in English (Oslo 2) where 53% of the respondents had taken their undergraduate studies outside of Norway.

The largest group of respondents were doing clinical research (45%), and 29% were doing basic research. 62% and 11% of the respondents had been a doctoral student less than one year and more than two years respectively. On average, the respondents in Tromsø had been doctoral students longer compared to respondents from the other universities.

59% of the respondents had attended lectures or courses in science ethics as part of their undergraduate studies while 31% had not.

The pilot study was carried out in Oslo including 26 respondents with a response rate of 55,3%.

To the questions about the experience of scientific dishonesty and other unethical behaviour in connection with research during the previous 12 months, on average 23% of the respondents answered that they had heard or read about researchers who had cheated (nationally or internationally), 65% had not heard about this, and 12% were uncertain. 29% had heard about fabricated data (see Table 2).

**Table 2:** Answers to questions about scientific dishonesty and other unethical behaviour in connection with research (Those who have answered YES in percent)

**Questions**	**Bergen**	**Oslo 1**	**Oslo 2**	**Tromsø**	**Trondheim**	**All Norway**	**All Sweden**
Have you, nationally or internationally, heard about anyone who during the last 12 months that has							
Fabricated data	21,1	28,3	33,3	36,7	29,3	29,2	29
Falsified data	18,4	23,9	23,3	30	24,4	23,8	31,8
Plagiarised data	13,2	19,6	20	23,3	29,3	21,1	24,2
Plagiarised publications	5,3	17,4	16,1	31,3	29,3	19,7	-
Have you yourself during the last 12 months been the object of pressure to							
Fabricate data	0	2,1	0	0	0	0,5	0
Falsify data	0	2,1	0	0	0	0,5	5,4
Plagiarise data	0	0	3,2	0	0	0,5	0
Plagiarise publications	0	0	0	0	0	0	-
Have you during the last 12 months been exposed to unethical pressure concerning							
Ordering of authors	13,2	8,7	12,9	12,5	7,3	10,6	8,5
Design/method	0	2,2	6,5	3,1	2,4	2,7	3,1
Results	0	0	12,9	0	2,4	2,7	0,8
Harassment	0	0	0	3,1	0	0,5	0,8
Have you during the last 12 months been affected by any consequences of scientific dishonesty							
Ethical	0	6,5	3,2	12,5	7,3	5,9	0
Legal	0	0	3,2	3,1	0	1,1	0
Methodological	0	4,3	0	3,1	7,3	3,2	-
Any other aspect	2,6	4,3	3,2	0	4,9	3,2	0

Data from Sweden reproduced from Nilstun 2010.

One respondent had experienced pressure to fabricate and to falsify data, and one had experienced pressure to plagiarize data, while none of the participants had felt pressure to plagiarize publications. Five respondents were uncertain if they had experienced pressure to fabricate data.

None of the respondents reported to have fabricated, falsified or plagiarized data or to have plagiarized publications. Only one respondent was uncertain whether he or she had plagiarized data and two were uncertain whether they had plagiarized publications.

About 11% of the respondents had experienced unethical pressure concerning the order of authors on publications during the last 12 months. In addition 7% stated that they didn’t know whether they have experienced such pressure. Five respondents had experienced unethical pressure concerning design/method and five had experienced unethical pressure concerning results, while one had experienced harassment.

About 6% of the respondents had been affected by ethical consequences of scientific dishonesty, and 3% had been affected by methodological consequences of scientific dishonesty during the last 12 months.

60% of the respondents were uncertain whether their department had a written policy about scientific conduct. 28% stated that their department had a written policy, and 12% that their department did not. Written policies on funding and on fabrication and falsification of data reached the highest reported awareness amongst the respondents (38%, 31%, and 31%). See Table 3.

**Table 3:** How many PhD-students were uncertain about whether their department had written policies (in percent)

**Questions**	**Bergen**	**Oslo 1**	**Oslo 2**	**Tromsø**	**Trondheim**	**All Norway**	**All Sweden**
Does your department have a written policy about							
Application for funds	63,9	44,7	48,4	62,5	53,7	54	59,2
Use of funds	63,9	36,2	41,9	65,6	61	52,9	57,7
Changes in design/method	72,2	63,8	61,3	77,4	68,3	68,3	47,3
Changes in results	77,8	59,6	71	77,4	65,9	69,4	43,1
Fabrication of data	75	38,3	48,4	71	61	57,5	44,6
Falsification of data	75	38,3	48,4	71	61	57,5	43,8
Ordering of authors	72,2	38,3	54,8	75	63,4	59,4	50,8
Plagiarism of others	75	46,8	48,4	71,9	53,7	58,3	49,6
Publishing the same twice	77,8	44,7	61,3	75	61	62,6	46,2
Harassment	83,3	53,2	41,9	68,8	65,9	62,6	43,8

Data from Sweden reproduced from [3].

The results on attitudes towards scientific misconduct (Table 4) show that 10% did not agree with the statement that it was never appropriate to report experimental data that have been created without actually having conducted the experiment, and 4% did not agree that it is never appropriate to alter experimental data to make an experiment look better than it actually was.

**Table 4:** **Proportion who answer that they *strongly agree *or *agree *with claims about actions and behavior in scientific research given in percent**

**Questions**	**Bergen**	**Oslo 1**	**Oslo 2**	**Tromsø**	**Trondheim**	**All Norway**
It is never appropriate to report experimental data that have been created without actually having conducted the experiment.	94,7	91,1	83,3	93,8	87,5	90,3
It is never appropriate to alter experimental data to make an experiment look better than it actually was.	100	93,5	90,3	100	97,5	96,3
It is never appropriate to try a variety of different methods of analysis until one is found that yields a result that is statistically significant.	68,4	47,8	51,6	73,3	71,8	62
It is never appropriate to take credit for the words or writing of someone else.	91,9	91,3	96,8	100	97,5	95,2
It is never appropriate to take credit for the data generated by someone else.	81,6	82,2	90,3	93,5	95	88,1
It is never appropriate to take credit for the ideas generated by someone else.	92,1	84,4	96,7	96,9	92,3	91,8
If you were confident of your findings, it is acceptable to selectively omit contradictory results to expedite publication.	8,1	14,3	23,3	12,9	7,7	12,8
If you were confident of your findings, it is acceptable to falsify or fabricate data to expedite publication.	2,6	17,8	6,5	13,3	10	10,3
It is more important that data reporting be completely truthful in a publication than in a grant application.	28,9	38,6	30	36,7	12,8	29,3
If you witness someone committing research misconduct, you have an ethical obligation to act.	81,6	80,4	96,8	87,1	92,5	87,1
If you had witnessed a co-worker or peer committing research misconduct, you would be willing to report that misconduct to a responsible official.	78,9	78,3	80,6	80,6	77,5	79
If you had witnessed a supervisor or principal investigator committing research misconduct, you would be willing to report that misconduct to a responsible official.	71,1	75,6	80	67,7	77,5	74,5
If fabricated data are discovered in a published paper, all co-authors must equally share in the blame.	60,5	28,3	51,6	48,4	45	45,7
If fabricated data are discovered in a published paper, all co-authors must receive the same punishment.	39,5	15,2	38,7	30	25,6	28,8

38% did not agree with the statement that it is never appropriate to try a variety of different methods of analysis until one is found that yields a result that is statistically significant, and 5% did not agree that it is never appropriate to take credit for the words or writing of someone else.

12% did not agree that it is never appropriate to take credit for data generated by someone else, and 8% did not agree that it is never appropriate to take credit for the ideas generated by someone else.

13% agreed that it is acceptable to selectively omit contradictory results to expedite publication and 10% found it acceptable to falsify or fabricate data to expedite publication, if they were confident of their findings, and 29% agreed that it is more important that data reporting be completely truthful in a publication than in a grant application. 13% agreed that you have an ethical obligation to act if you witness someone committing research misconduct.

79% agreed they would be willing to report that misconduct to a responsible official, if they had witnessed a co-worker or peer committing research misconduct, and 75% agreed that they would be willing to report misconduct to a responsible official if they had witnessed a supervisor or principal investigator committing research misconduct.

46% agreed that all co-authors must equally share in the blame if fabricated data are discovered in a published paper, and 29% agreed that all co-authors must receive the same punishment if fabricated data are discovered in a published paper.

There were some differences between the universities. E.g., only half of the PhD-students in Oslo disagreed that it was never appropriate to try a variety of different methods of analysis until one is found that yields a result that is statistically significant. More PhD-students at the course in English in Oslo (Oslo 2) than the average found it acceptable to selectively omit contradictory results to expedite publication if they were confident of their findings. Fewer PhD-students at Oslo 1 and more in Bergen agreed that all co-authors must equally share in the blame if fabricated data are discovered in a published paper than the average. Fewer of the PhD-students in Trondheim agreed that it is more important that data reporting be completely truthful in a publication than in a grant application than the average. In Tromsø fewer respondents than the average reported that it is never appropriate to try a variety of different methods of analysis until one is found that yields a result that is statistically significant. More PhD-students with undergraduate studies outside Norway answered that they had been exposed to unethical pressure concerning results during the last 12 months than those who had studied in Norway (p = 0.045, Fischer’s exact test), and that they were uncertain whether they had been exposed to unethical pressure concerning harassment (p = 0.02, Fischer’s exact test). The PhD-students with undergraduate studies outside Norway differed also from those that had studied in Norway in that they found it more acceptable to selectively omit contradictory results to expedite publication if they were confident of their findings (p = 0.008, Mann–Whitney).

## Discussion

The findings show that scientific dishonesty is not unknown to Norwegian doctoral students and that they found some actions acceptable which are considered to be misconduct in the science ethics literature. There was little awareness of relevant policies for scientific conduct, but a high level of willingness to report misconduct.

### Awareness of policies

Many of the respondents were unaware of their departments’ policies concerning scientific conduct. The reason for this may be due to organizational distances between science ethics policy makers and researchers (and perhaps even more research students), and indicates that communication about such policies needs to be improved and implemented better in order to work as intended. Another reason may be that many scientists are uncomfortable to talk about research misconduct and reluctant to act when they see it [27]. If this is so, there is an urgent need to enhance awareness, procedures and measures [28]. Moreover, as recognized by Nilstun [3], there are two interpretations of those doctoral students who were uncertain about whether there existed a policy at their department: “The first interpretation is that the respondents are certain that a policy exists, but they are uncertain about what it prescribes. The second interpretation is that the respondents are uncertain as to whether or not a policy exists at all”. As Nilstun and colleagues point out, both interpretations raise challenges to present policies.

The Sudbø case can explain the difference between the results in Norway and Sweden. Moreover, the University of Oslo elaborated and put great emphasis on ethical guidelines at all of its faculties. This was not done in the same manner at the other universities in Norway, who do not have specific guidelines or policies, but who refer to national legislation.

### Pressure and reporting

About 11% of the respondents in Norway had experienced unethical pressure concerning the order of authors. In addition 7% answered that they don’t know whether they have experienced unethical pressure concerning the order of authors. It may well be that these 7% have experienced some kind of pressure, but were unsure whether this experience qualified for the label “unethical” or not. If you haven’t experienced the order of authors as an issue at all, then you would probably answer “No” rather than “Don’t know”. Combined this means as much as 18% *may have* experienced unethical pressure concerning the order of authors.

The results also show that a considerable number of PhD-students experience pressure towards other kinds of scientific dishonesty, which is worrying. It indicates that scientific dishonesty has to be further addressed at an organizational level and not only at the level of the individual researcher [17].

More positive is that most participants state that they would report scientific misconduct if they experienced it.

### Comparison with other surveys

Our results are in accordance with the results from the Swedish study [3]. This may be because the cultural differences between the Scandinavian countries are small, because there is extensive scientific collaboration and exchange between the countries, and because the educational system is similar. The results tentatively indicate less scientific dishonesty in Norway than in other countries [9,11]. This counters previous results from Norway, showing that 22% of 274 medical scientists knew about cases of serious misconduct, 9% had themselves contributed to one or more incidents of misconduct, and 3% of the respondents were aware of falsification or fabrication of data [29]. However, the Swedish and our survey asked about scientific dishonesty specifically within the last 12 months, whereas other studies were less specific and did not have this time restraint. If respondents have answered with a view to the last 12 months in our study (and in the study of Nilstun et al.) then our results should be expected to be lower than in studies reporting what is essentially a life time prevalence rate, which from a scientist’s perspective is considerably more than 12 months.

No participants in this study reported that they had fabricated, falsified or plagiarized data or plagiarized publications, while four persons in the Swedish survey reported fabrication or falsification of data [3]. However, three persons in the Norwegian study were uncertain whether they had plagiarized data or publications (compared to three persons who were uncertain about plagiarizing data in the Swedish survey). The low rates are in line with previous studies [30,31], but it may of course be that participants are cautious concerning reporting scientific dishonesty to a study performed by researchers at their faculty who teach science ethics, even when the answers are anonymous. Hence, the method may not be adequate to reveal scientific dishonesty, or the presupposition that we only discover the top of the iceberg [27] may be wrong. However, the low rates and the ‘unsure’ answers to whether respondents had falsified or plagiarised data fits well with a lax conception of scientific dishonesty. People with a lax conception of falsification or plagiarism, will not report to have falsified or plagiarized data in the same situation where others would have reported to have done so or that they were unsure.

When comparing with other countries it is also worth noting that the Nordic countries define scientific dishonesty slightly differently [23]. The current Swedish guidelines for judging alleged scientific fraud state that the state of affairs—not the intention—is important [32] whereas intentions are relevant in Norway, e.g., for the assessment of the severity of the misconduct [31].

Although results from studies with undergraduate students from various parts of the world are relevant [33-35], it may be challenging to compare them to results from PhD-students.

### Types of misconduct

A relatively large proportion of the respondents found some actions acceptable which are considered to be misconduct in the science ethics literature. E.g., responses from 11 of the 189 respondents (5,8%) indicate that they think it can be appropriate to report experimental data that have been created without actually having conducted the experiment and 20 respondents (10,6%) indicated that it is appropriate to try a variety of different methods of analysis until one is found that yields a result that is statistically significant. 23 participants (12,2%) agreed that it is acceptable to selectively omit contradictory results to expedite publication and 19 (10,1%) found it acceptable to falsify or fabricate data to expedite publication, if they were confident of their findings. This corresponds with other studies [6,7,15].

At the same time most of the participants would report research misconduct if they witnessed it, even if it was committed by the principal investigator or their supervisor. Whether they would do so in actual cases, we do not know, but it is interesting that the respondents appear to have rather lax conceptions of scientific misconduct, but that they are willing to report it if they saw it. The reason for this may be that revealing misconduct, such as in the Sudbø case, can be meriting. There is hence no necessary connection between willingness to report misconduct and a strict conception of misconduct.

In Norway about 6% of the respondents had been affected by ethical consequences of scientific dishonesty, and 3% by methodological consequences of scientific dishonesty in the last 12 months, compared to none in Sweden. The reason for this may be that some large research projects in Norway were stopped or altered recently due to scientific dishonesty, which had severe consequences for the PhD-students on these projects.

The results underscore the necessity for efficient measures to reduce misconduct. However, the effectiveness of the organisational procedures for reducing misconduct has been reported to be lower in medical centres than in other settings [36]. Training and attending courses may not be the only or the best way to go [8]. Supervisors and principal investigators may need more awareness [6,37], as higher incidents of misconduct has been observed among mid-career scientists who often are supervisors for PhD candidates.

### Methodological limitations

There are methodological limitations in relation to the questionnaire. No validated questionnaires were available, but a questionnaire that had previously been applied (and published) was used in order to enable direct comparison. The face validity of the Swedish questionnaire was tested and our pilot study resulted in an improved survey introduction and some additional questions. Ideally we would have made a more thorough validation of the questionnaire and its reliability, for instance through a test re-test procedure. However, the setting with the PhD-courses does not easily permit this. It appears to be an advantage to our study that we compare the respondents’ reported actions with their attitudes as which was not done in the Swedish study. The response rate in our study is higher than in the Swedish study (71.2% versus 58%). We obtained the highest response rate when time was allocated to filling in the form. Longer time between handing out and handing in the form resulted in lower response rate.

Although there are reasons to believe that at least some PhD-students have cheated before (during their undergraduate studies), there appear to be cultural differences in attitudes to teaching [20]. For example it has been reported that at the University of Oslo several of the students at previous PhD-courses have been caught in copying at exams (even when signing a written statement concerning referencing and plagiarism) [38]. It turned out that most of these students were trained in countries with different scientific norms. Our study did not reveal great difference between those attending the English-speaking course and the Norwegian-speaking course in Oslo (Oslo 1). The only question where there was a noteworthy difference was whether the respondent found it acceptable to selectively omit contradictory results to expedite publication if they were confident of their findings, where 23% of the participants at the English-speaking course (Oslo 2) agreed, compared to 14% of the participants at the Norwegian-speaking course (Oslo 1) and 13% in all Norway.

Although our study showed some substantial differences between the PhD-students who had their undergraduate studies in and outside Norway, this is not beyond what one could expect to find by pure randomness.

Although it is argued that ethics training and mentoring is not effective in preventing scientific dishonesty [15], and that dishonesty may be related to personality traits [39], there is a wide range of strategies to avoid scientific dishonesty in medical research available [40]. Moreover, important measures to avoid and reveal scientific dishonesty are taken by associations of journals, such as World Association of Medical Editors (WAME), Council of Science Editors (CSE), European Association of` Science Editors (EASE), and Committee on Publication Ethics (COPE). More research is needed to find the most effective measures to reduce scientific dishonesty.

## Conclusion

The survey shows that scientific dishonesty is not unknown to PhD students in Norway. Very few stated that they were exposed to pressure to fabricate, falsify, or plagiarise data, while pressure put upon post graduate students regarding the order of authors was more common. Some forms of scientific misconduct were considered to be acceptable by a substantial minority. e.g., almost two of five respondents found data fishing acceptable. There was little awareness of relevant policies for scientific conduct, but a high level of willingness to report misconduct.

With the intention to build more awareness and improved attitudes towards various forms of scientific dishonesty our suggestions are:

• Increase the quality of teaching, use cases, focus on grey areas, and communicate the norms of good scientific practice repeatedly and in a variety of settings.

• Provide better training for supervisors and ensure that they have the appropriate knowledge and promote attitudes.

• Clarify institutional policies towards scientific dishonesty and communicate them more effectively.

## Competing interests

All authors are involved in teaching science ethics to PhD-students.

## Authors’ contributions

Søren Holm (SH) and Bjørn Hofmann (BH) elaborated the questionnaire which was revised by all authors. All authors participated in data collection. BH performed data analysis and developed the first draft of the manuscript. All authors contributed significantly to the manuscript and its revision and have approved the final version.

## Pre-publication history

The pre-publication history for this paper can be accessed here:

http://www.biomedcentral.com/1472-6939/14/3/prepub
